# The Apostles Creed

**Published:** 1880-12

**Authors:** 


### THE APOSTLES CREED.

The following very clever hit at the scientific unbelief of the day, written by Mr. A. Bierbower, of this city, appears in the last issue of the "New York Independent:"

"I believe in a chaotic nebula, self-existent, evolver of heaven and earth, and in the differentiation of the original homogeneous mass, its first begotten product, which was self-formed into separate worlds, divided into land and water, self-organized into plants and animals, reproduced into like species, further developed into higher orders, and ultimately refined, rationalized, and perfected in man. He descended from the monkey, ascended to the philosopher, and sitteth down in the rights and customs of civil. zation under the laws of a developing sociology. From thence, he shall come again, by the disintegration of the heterogenized cosmos back to the original homogeneousness of chaos.

"I believe in the wholly impersonal absolute, the wholly un-catholic church, the disunion of the saints, the survival of the fittest, the persistence of force, the dispersion of the body, and its death everlasting."

